# 492. Risk Factors for COVID-19 Mortality in ≥90 year old Inpatients: Retrospective Case Series

**DOI:** 10.1093/ofid/ofad500.561

**Published:** 2023-11-27

**Authors:** Raphaela Martina C Pineda, Monica Castro, Gerald Kristoffer E Boy, Sushil Pant

**Affiliations:** Mount Sinai, New York, New York; UERM, Tempe, Arizona; Montefiore New Rochelle Hospital, New York City, New York; Montefiore New Rochelle Hospital, New York City, New York

## Abstract

**Background:**

Despite what we know about COVID-19, current literature is still lacking on some aspects of the disease. This is probably because the disease continues to evolve and there is still much to discover. COVID-19 outcomes in the elderly is one aspect that has not been fully explored.

This study investigated short-term outcomes of COVID-19 Infection on ≥90 year old patients in the context of COVID vaccination availability. We sought to identify risk factors for in-patient all-cause mortality during the peak of daily COVID-19 infections in this population.

**Methods:**

52 admitted patients aged ≥90 years old with SARS-CoV-2 RT-PCR positive observed from Nov 2021 to Mar 2022 at New Rochelle Hospital in NY, USA.

We conducted a retrospective case study of ≥ 90 years old patients admitted at New Rochelle Hospital in NY, USA from Nov 2021 to Mar 2022. Electronic health records were reviewed.

Logistic regression was used to determine odds ratio for risk factors that contribute to COVID-19 mortality. Univariate analysis was used to analyze patients’ comorbidities, symptoms, lab abnormalities, and imaging with mortality as the independent variable.

**Results:**

The most frequent risk conditions for COVID-19 mortality are congestive heart failure (p< 0.048) and atrial fibrillation (p< 0.009). The odds of dying from COVID19 is 5 times greater for nursing home residents than those patients residing at home or already admitted in the hospital. Shortness of breath and fever were found to be statistically significant with COVID 19 mortality. Patients who had x-ray results typical for COVID (p< 0.004), leukocytosis (p< 0.039), acute kidney injury (p< 0.003), and elevated transaminases (p< 0.001) are more at risk for dying from COVID.

**Conclusion:**

Managing elderly individuals with COVID-19 can be challenging due to increased risk of severe illness and complications associated with age. We found that certain risk factors such as co-morbidities, previous residence, presenting symptoms and laboratories are associated with increased mortality in patients ≥ 90 years old. Currently, limited guidelines and studies are available for the geriatric population. Further research can be done by stratifying severity of comorbidities, determining vaccination status, and increasing sample size.

**Disclosures:**

**All Authors**: No reported disclosures

